# Supplementary material for: Functional Evaluation of Splicing for Variants of Uncertain Significance in Patients with Inherited Retinal Diseases
Source: Genes (Basel). 2021 Jun 29;12(7):993. doi: 10.3390/genes12070993 (PMC8303747; doi:10.3390/genes12070993)
Supplement: Supplementary file 1 [file genes-12-00993-s001.zip › genes-1273228-supplementary.pdf]

Table S1. Constructs for minigene assays

Gene	Transcript Accession Number	Nucleotide Variant (location)	Primers	Exons in construct	Genomic fragment size in bp
OPA1	NM_130837.2	c.2797G>T (exon 28)	F: GCAGAATTTCATGGCTCCGT<br>R: GGAGTCCATTCCCAACAGAG	Exon 28	801
CNGB1	NM_001297.4	c.2492+1G>A (intron 25)	F: ATCCGATGCCCCTCATTGTG<br>R: GCAGGTGCTCAGGAAACCTT	Exon 25	1187
		c.583+2T>C (intron 9)	F: CCTGGGAACATGGTGCTTCT<br>R: GCTCCTGTCAGGTGTGTGTT	Exons 8-10	1820
		c.2305-34G>A (intron 23)	F: GTGGGAGTGTCCTCCAAACC<br>R: AGGTCAGTGGGGGTAGACAA	Exons 23-24	3489
CLUAP1	NM_015041.3	c.929-142G>A (intron 9)<br>C.930T>A (exon 10)	F: GGCGAGAGAGAGCAAGAG<br>R: GCTTTGTTGCTTGAATCTTTTGTG	Exon 10	972

Table S2. In silico analysis of the identified variants altering Non-Canonical Splice Sites (NCSS), Gene, Location and Minimum Allele Frequency (MAF) of each variant is indicated. Relevant changes are highlighted in red.

Gene	Variant	SSF				MaxEntScan				NNSPLICE				GeneSplicer				EX-SKIP ESS/ESE		EX-SKIP	Align GVD (v2007)	SIFT (v6.2.0)	MutationTaster (v2013)
		WT 5'	MUT 5'	WT 3'	MUT 3'	WT 5'	MUT 5'	WT 3'	MUT 3'	WT 5'	MUT 5'	WT 3'	MUT 3'	WT 5'	MUT 5'	WT 3'	MUT 3'	WT	MUT				
OPA1	c.2797G>T (p.Val933Phe)	NR	NR	79	78.9	NR	NR	5.3	5.34	NR	NR	1	0.57	NR	NR	1	1.1	1.9	2.44	MUT allele has higher chance of exon skipping	C0 (GV: 353.86 - GD: 0.00)	Deleterious (score: 0.01, median: 3.33)	disease causing (prob: 1)
CNGB1	c.2492+1G>A	90	NR	NR	NR	9.6	NR	3	1.8	1	NR	NR	NR	7.4	NR	NR	NR	NA	NA	NA	NA	NA	NA
	c.2305-34G>A	71	NR	NR	70.2	6.2	NR	NR	1	NR	NR	NR	NR	1.9	NR	NR	NR	NA	NA	NA	NA	NA	NA
	c.583+2T>C	72	NR	NR	NR	8.7	NR	NR	NR	0.9	NR	NR	NR	10	NR	NR	NR	NA	NA	NA	NA	NA	NA
CLUAP1	c.930T>A (p.Ser310Arg)	78	NR	82	82.2	7.7	NR	11	9.6	0.7	NR	1	0.5	NR	NR	12	11	0	0	Both alleles have equal chance of exon skipping	C0 (GV: 250.76 - GD: 19.25)	Tolerated (score: 0.39, median: 3.58)	disease causing (prob: 1)
	c.929-142G>A	NR	NR	NR	79.4	NR	NR	NR	5.6	NR	NR	NR	NR	NR	NR	NR	NR	NA	NA	NA	NA	NA	NA

Not applicable (NA); SSF: splice site finder; WT: wild type; MUT: mutant; NR: not recognized; Cutoff SSF 70;
